# Segregation of Hydroxycinnamic Acid Esters Mediating Sweetpotato Weevil Resistance in Storage Roots of Sweetpotato

**DOI:** 10.3389/fpls.2017.01011

**Published:** 2017-06-13

**Authors:** Milton O. Anyanga, Benard Yada, G. C. Yencho, Gorrettie N. Ssemakula, Agnes Alajo, Dudley I. Farman, Robert O. M. Mwanga, Philip C. Stevenson

**Affiliations:** ^1^National Crops Resources Research Institute, National Agricultural Research OrganizationKampala, Uganda; ^2^Department of Agriculture Health and Environment, Natural Resources Institute, University of GreenwichChatham, United Kingdom; ^3^Department of Horticultural Science, North Carolina State University, RaleighNC, United States; ^4^International Potato CenterKampala, Uganda; ^5^Natural Capital and Plant Health, Royal Botanic Gardens, KewRichmond, United Kingdom

**Keywords:** sweetpotato weevil, insect resistance, storage root chemistry, segregating population, *Cylas*

## Abstract

Resistance to sweetpotato weevils (*Cylas* spp.) has been identified in several sweetpotato (*Ipomoea batatas*) landraces from East Africa and shown to be conferred by hydroxycinnamic acids that occur on the surface of storage roots. The segregation of resistance in this crop is unknown and could be monitored using these chemical traits as markers for resistance in F_1_ offspring from breeding programs. For the first time in a segregating population, we quantified the plant chemicals that confer resistance and evaluated levels of insect colonization of the same progeny in field and laboratory studies. We used a bi-parental mapping population of 287 progenies from a cross between *I. batatas* ‘New Kawogo,’ a weevil resistant Ugandan landrace and *I. batatas* ‘Beauregard’ a North American orange-fleshed and weevil susceptible cultivar. The progenies were evaluated for resistance to sweetpotato weevil, *Cylas puncticollis* at three field locations that varied climatically and across two seasons to determine how environment and location influenced resistance. To augment our field open-choice resistance screening, each clone was also evaluated in a no choice experiment with weevils reared in the laboratory. Chemical analysis was used to determine whether differences in resistance to weevils were associated with plant compounds previously identified as conferring resistance. We established linkage between field and laboratory resistance to *Cylas* spp. and sweetpotato root chemistry. The data also showed that resistance in sweetpotato was mediated by root chemicals in most but not all cases. Multi-location trials especially from Serere data provided evidence that the hydroxycinnamic acid esters are produced constitutively within the plants in different clonal genotypes and that the ecological interaction of these chemicals in sweetpotato with weevils confers resistance. Our data suggest that these chemical traits are controlled quantitatively and that ultimately a knowledge of the genetics of resistance will facilitate management of these traits, enhance our understanding of the mechanistic basis of resistance and speed the development of new sweetpotato varieties with resistance to sweetpotato weevil.

## Introduction

Sweetpotato (*Ipomoea batatas* (L.) Lam) is a globally important crop (Scott et al., 1999; Andrade et al., 2009). It is particularly important in sub-Saharan Africa where it contributes to food security and income generation for marginalized farmers. Damage caused by insects is a major constraint to sweetpotato production. Sweetpotato weevils (SPW) (*Cylas* spp.), in particular, are very damaging as they attack vines and roots, resulting in an unacceptable odor and bitter taste, rendering the roots unfit for human and animal consumption (Uritani et al., 1975) and can cause crop losses of up to 98% (Smit, 1997; Nottingham and Kays, 2002). The concealed subterranean and within root feeding behavior of SPW severely complicates their management and control (Nottingham and Kays, 2002; Odongo et al., 2003). The major damage reported is caused by feeding from both SPW adults and larvae which tunnel inside the storage roots and induce production of sesquiterpenes that appear not to affect the insect but make it unfit for sale or human consumption (Uritani et al., 1975; Sato and Uritani, 1981). The two indigenous African species, *Cylas puncticollis* and *C. brunneus*, can co-infest the same plant and develop in the same root. Farmers typically use cultural practices such as field sanitation, hilling up and timely planting and harvesting before drought to enable escape but the current practice is not cost effective or consistently implemented (Stathers et al., 2003).

Host plant resistance is therefore likely to provide a major component of any integrated pest management (IPM) program for sweetpotato. However, the development of weevil resistant varieties has not been successful owing to a lack of heritable resistance present in the sweetpotato germplasm evaluated over years.

Recently, Muyinza et al. (2012) reported that the gene pool of African landraces and varieties of sweetpotato was highly heterogeneous in their susceptibility to field infestations of SPW with several showing resistance. Similar levels of resistance under laboratory conditions indicated that resistance was an active mechanism and not simply a consequence of escape through field phenology such as deep rooting plant phenotypes which tend to avoid SPW damage (Stathers et al., 2003). Stevenson et al. (2009) identified hydroxycinnamic acid esters in the root latex of sweetpotato resistant to SPW. These compounds reduced development of SPW larvae and suggested that differences in the concentration of these compounds between varieties might explain differences in resistance. Anyanga et al. (2013) reported that the occurrence of these compounds within root cortex did not differ significantly among resistant and susceptible varieties but that their presence in high concentrations on root surfaces was strongly associated with resistance with a strong effect against adult oviposition and feeding.

Hydroxycinnamic acid esters were also associated with resistance to *C. formicarius* in sweetpotato genotypes from the United States (Snook et al., 1994; Data et al., 1996) thus these components may provide globally relevant traits to confer resistance to all major *Cylas* species. Hydroxycinnamic acids are a major group of phenolic acids with bioactive properties that are common in the plant kingdom (Levin et al., 2010). They are produced for protection against biotic and abiotic stress (Heleno et al., 2015). According to Xu et al. (2009), hydroxycinnamic acids are a product of the biochemical pathway that yields lignin, the polymeric material that provides mechanical support to the plant cell wall. To fully understand the potential of this resistance mechanism, however, it was necessary to study the segregation pattern in a population of a cross between a resistant and a susceptible variety. This would pave the way for research aimed at combining the resistance with key agronomic traits.

The objective of this study was to determine the segregation pattern of resistance to SPW in a bi-parental cross between New Kawogo and Beauregard. Association analyses of SPW resistance and SSR markers was recently reported for this same population (Yada et al., in press) and a genetic map based on SNP markers is currently being developed for this population under the Genomic Tools for Sweetpotato Improvement (GT4SP) project (Yencho, unpublished) with the overarching aim of facilitating the identification of quantitative trait loci associated with sweetpotato weevil resistance.

## Materials and Methods

### Plant Material

An F_1_ population of 287 progenies from a bi-parental cross between an African landrace that is resistant to SPW, *I. batatas* ‘New Kawogo’ (NK) (Stevenson et al., 2009; Muyinza et al., 2012; Anyanga et al., 2013) and a susceptible North American orange-fleshed variety *I. batatas* ‘Beauregard’ (B), was generated at the National Crops Resources Research Institute (NaCRRI), Namulonge, Uganda. The *I. batatas* ‘New Kawogo’ (female parent) is of particular interest as a progenitor of the segregating traits since it is not only resistant to SPW but also has field resistance to sweetpotato virus disease (SPVD). NK is white-fleshed and also has high dry matter compared to Beauregard (Mwanga et al., 2001, 2003, 2007). Beauregard (male parent) is a SPW and SPVD susceptible variety, is orange-fleshed and is a popular variety in North America (Rolston et al., 1987).

### Evaluation of Weevil Resistance in Field Experiments

The 287 progeny and the two parents were evaluated for SPW resistance at three Ugandan agricultural research station sites, Serere, Namulonge, and Ngetta for two seasons (**Table 1**). All the experiments were planted in a randomized complete block design (RCBD) with three replications per genotype per site on 1.5 m ridged plots following the procedure used by Muyinza et al. (2012). Five plants, each from a 30 cm vine cutting of every progeny (genotype), were planted in plots at a spacing of 30 cm between plants and the ridges were separated by a 1.0 m walk way.

**Table 1:** Experimental sites.

Location	GPS coordinates	Agro-ecological zone	Average daily temperature
NaCRRI, Namulonge	0° 32′N, 33° 35′E, 1,160 masl	Moist tall grassland	27°C
NaSARRI, Serere	1° 32′N, 3° 27′E, 1,085 masl	Dry, short grassland	31.3°C
NgeZARDI, Ngetta	2° 202′ N, 33° 62′E, 1,080 masl	Dry, short grassland	30°C

Each plot was artificially infested with 10 *C. puncticollis* and *C. brunneus* in a ratio of 7 female to 3 male SPW at 90 days after planting (DAP) to augment the natural weevil population and ensure effective exposure using procedures adopted from earlier work reported by Muyinza et al. (2012). At 90 DAP the storage roots are already formed and capable of causing soil cracking to expose the roots. The artificial infestation helped build up the existing weevil population to damaging levels that can enhance evaluation of the genotypes as susceptible or resistant. The weevils used in the trials were reared on a SPW susceptible variety NASPOT 1 using procedures developed by Stevenson et al. (2009). The first season trials were planted in June and harvested in November 2012. The second season trials were planted in November, 2012 and harvested in May, 2013. The harvesting of both trials was done at 5 months after planting (MAP), by this time any SPW would have caused sufficient damage to allow for the evaluation of sweetpotato varieties as either resistant or susceptible.

### SPW Stem and Root Damage in Field Experiments

Plant vine weight and the number of infested storage roots was recorded at harvest. SPW storage root damage was assessed by estimating the percent damage inflicted on roots. Three vines were sampled per plot. Stem base damage was assessed by cutting the first 10-15 cm from two of the three vines per plot and rating damage following the 1–5 scale used by Muyinza et al. (2012) for evaluating stem base SPW damage where 1 = 0–20% of the basal segment damaged; 2 = 21–40%; 3 = 41–60%; 4 = 61–80%, and 5 = 81–100%. Storage root damage was assessed by counting the number of infested storage roots, cutting out infested portions with a kitchen knife and estimating the percentage infestation using the formula:

% damage =weight of clean roots−weight of chopped portion of infested/weight of whole root per plot×100

where 100% indicated that the storage root was totally damaged by SPW.

### Analysis of Total Hydroxycinnamic Acid Esters in Root Samples

Fresh roots of the 287 different progenies obtained from the field sites located at Ugandan Agricultural Ministry Research Stations: Ngetta Zonal Agricultural Research and Development Institute (NgeZARDI), National Semi Arid Resources Research Institute, Serere (NaSARRI) and National Crops Resources Research Institute, Namulonge (NaCRRI) and the two parents were cleaned of loose soil and left to air dry at room temperature. Fresh roots were cut transversely into 2–3 root disks from the middle portion of each root sample, weighed, packed in polythene bags and freeze-dried using a vacuum freeze dryer (True-Ten Industrial Co., Taichung City, Taiwan) for 72 h. The root surface of the freeze-dried disks was separated from the disk using a pair of pliers and blended into powder. Fifty milligrams of the powder was weighed using a Mettler 2001 weighing scale and placed in an Eppendorf tube. Methanol (1 ml) was placed onto the sample in the Eppendorf tube and extracted for 24 h. The sample was centrifuged and the filtrate in methanol (1 ml) used for the phytochemical analysis using liquid chromatography-mass spectrometry (LC-MS) to quantify hydroxycinnamic acid esters following the procedures used by Anyanga et al. (2013).

### Evaluation of Feeding and Oviposition Bioassay

Sweetpotato weevil feeding and oviposition were observed on the storage roots of individual progenies inoculated with 10 two week old gravid female *C. puncticollis* adults for 24 h to feed and lay eggs. The roots from the field experiment were placed in plastic bottles with the top covered with muslin cloth for aeration. The weevils were removed after 24 h and the number of feeding holes on the roots were counted and recorded. The eggs laid in the roots during the time of weevil exposure were left to incubate until emergence. After artificial infestation, the number of adults emerging was recorded from day 25 to day 50 every 5 days.

### Data Collection and Analysis

Data analysis was performed on only 284 different offspring and the parents. Three progeny were dropped because their field establishment was poor with 2–3 plots dead in all the three sites making them unsuitable for analysis. The data for the two seasons were pooled for analysis. Field data were pooled across seasons and locations. Analysis of variance (ANOVA) of sweetpotato field resistance data was conducted using R statistics with Statistical Services Centre, University of Reading, United Kingdom. Genotypes were treated as fixed effects and block, site and season as random effects. The genotype means were used to assess the level of transgressive segregation for sweetpotato weevil resistance. For total hydroxycinnamic acid ester concentration, ANOVA using linear and Pearson correlation analysis of field sweetpotato weevil infestation and hydroxycinnamic acid ester production was done using R statistics.

## Results

### Evaluation of Field Weevil Damage

**FIGURE 1:**
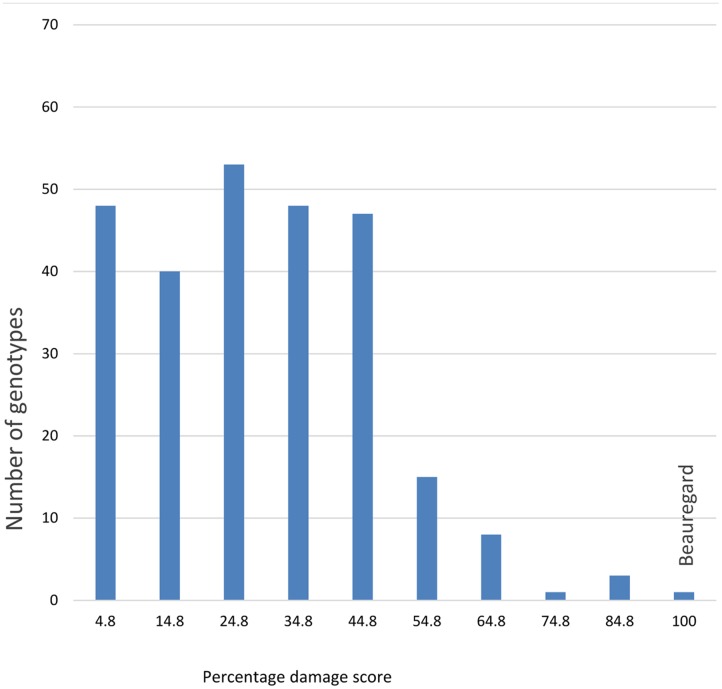
Number of plants showing segregation of resistance at each percentage damage score of sweetpotato weevil in the segregating population of *Ipomoea batatas* ‘New Kawogo’ and ‘Beauregard’.

**FIGURE 2:**
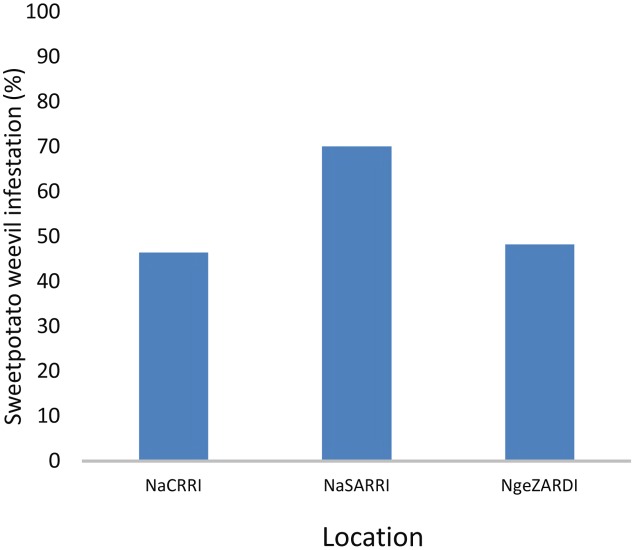
Mean (% ± standard error of the mean) sweetpotato weevil root infestation of the segregating population of *I. batatas* ‘New Kawogo’ and ‘Beauregard’ in three locations in Uganda.

**FIGURE 3:**
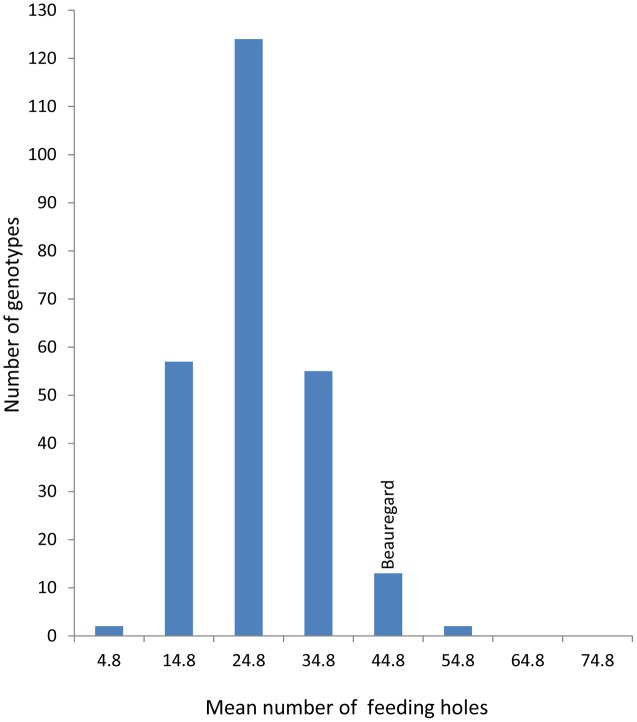
Mean number of feeding holes in a segregating population of *I. batatas* ‘New Kawogo’ (NKB288) and ‘Beauregard’ (NKB289).

**FIGURE 4:**
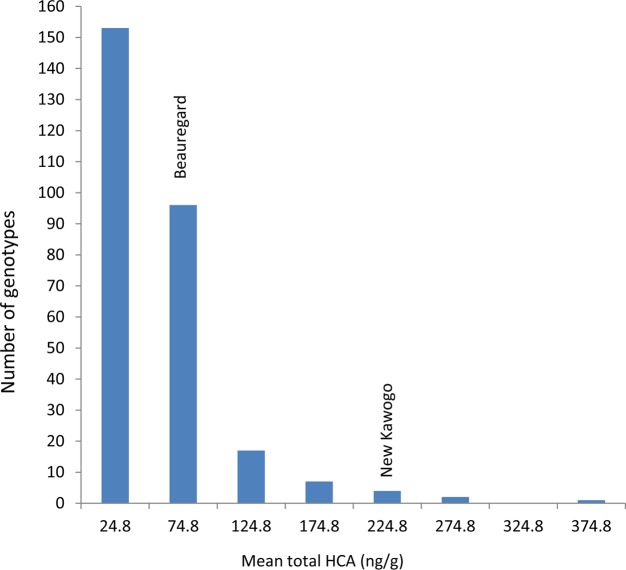
Mean total hydroxycinnamic acid (ng/g) in the segregating population of New Kawogo (NKB288) and Beauregard (NKB289).

Significant differences (ANOVA, *P* ≤ 0.001) were observed in the percentage of SPW infesting storage roots among locations and for genotypes of the segregating population (**Figures 1–4**). Twenty five of the 284 genotypes recorded storage root damage of less than 10% weevil infestation. The genotypes with low storage root SPW infestation included New Kawogo which was the resistant female parent in the cross. ‘Beauregard’ the susceptible male parent of the cross on the other hand, had mean storage root infestation of 100%, indicating that all storage roots showed weevil damage. The rest of the genotypes had varying root infestation between that of New Kawogo and Beauregard (**Figure 1**). There was a significant spatial effect (ANOVA, *P* ≤ 0.001) on sweetpotato weevil infestation of the genotypes as the mean sweetpotato weevil infestation was higher at NaSARRI than the mean root infestation at NaCRRI and Ngetta ZARDI (**Figure 2**).

The effect of genotype by season interaction was significant (ANOVA, *P* ≤ 0.001) on SPW root infestation. Mean SPW storage root infestation was significantly higher (ANOVA, *P* ≤ 0.05) in the experiment planted in season 2 than the infestation observed in season 1.

The genotype × environment (G × E) interaction effect was also significant (ANOVA, *P* ≤ 0.001) for SPW damage on the stem portion of the sweetpotato vine suggesting that weevil stem damage is climate dependent. Mean SPW stem damage was higher in season 1 (November to May) than in season 2 (June to November) (**Figure 4**).

There was a significant difference (ANOVA, *P* ≤ 0.001) in SPW stem damage on the sweetpotato vine in the three locations. The mean stem damage score on the sweetpotato vine was higher at NaSARRI, with internal stem damage score of 2.3 and 2.3, than it was at Ngetta ZARDI and NaCRRI where damage in the order of 1.5 and 1.6 and 1.4 and 1.5 was recorded for seasons 1 and 2, respectively.

### Effect of the Different Progenies on Feeding and Oviposition of SPW

The mean number of SPW feeding holes differed significantly (ANOVA, *P* ≤ 0.001) on the storage roots of the 284 genotypes of the segregating population. Fifty seven genotypes had a mean number of less than 20 feeding holes on the storage root (**Figure 3**) indicative of resistance while for susceptible varieties feeding damage was more than double this value. The mean number of *C. puncticollis* feeding holes on the roots of New Kawogo (NKB288) and Beauregard (NKB289) were 29 and 42, respectively (**Figure 3**). NKB152, NKB257, NKB72, NKB59, NKB260, NKB225, NKB108, NKB52, NKB158, and NKB279 had the lowest mean number of feeding holes caused by *C. puncticollis*.

### Phytochemical Analysis of the Segregating Population

There was a significant difference (ANOVA, *P* ≤ 0.001) in total hydroxycinnamic acids (HCA) on the root surface among the genotypes (progeny and parents) of the segregating population. The mean total HCA esters on the root surface of New Kawogo and Beauregard were 282 and 70.4 ng/g, respectively. Genotype mean total HCA ester concentrations ranged from 4.9 to 366.5 ng/g, in genotype NKB175 and NKB257, respectively. The distribution of genotype mean total HCA ester concentration was skewed to the left and only one progeny NKB257 had higher total HCA ester concentration than New Kawogo, the resistant parent (**Figure 4**). Over 70% of the progeny had less than 100 ng/g mean total HCA ester concentrations; an indication of low levels of SPW resistance (**Figure 4**). There was significant positive correlation between the total HCA ester concentration in a genotype and SPW field infestation (*r* = 0.603, *P* = 0.015).

The 10 genotypes with highest mean total HCA ester concentration were: NKB257 (366.5 ng/g), NKB152 (357.5 ng/g), NKB288 (282 ng/g), NKB108 (268.9 ng/g), NKB256 (254.0 ng/g), NKB265 (237.1 ng/g), NKB100 (228.8 ng/g), NKB258 (205.1 ng/g), NKB254 (185.5 ng/g), NKB59 (183.5 ng/g), and NKB60 (181.5 ng/g). The genotypes that exhibited the lowest concentration of mean total HCA esters were: NKB175 (4.9 ng/g), NKB285 (5.0 ng/g), NKB182 (6.3 ng/g), NKB29 (8.1 ng/g), NKB223 (8.7 ng/g), NKB138 (8.8 ng/g), NKB195 (9.2 ng/g), NKB28 (10.3 ng/g), NKB8 (11.9 ng/g), and NKB68 (12.1 ng/g).

Out of the top 10 progenies with high mean total HCA ester concentrations and sweetpotato root infestation, only five genotypes (NKB152, NKB257, NKB108, NKB59, and NKB60) showed high consistent performance on the basis of SPW root infestation and total HCA ester concentrations.

The effect of location was significant (*P* ≤ 0.001) for total storage root HCA esters. The mean total HCA esters were higher for storage roots from the experiments planted at NaSARRI an area with higher weevil infestation than on sweetpotato roots planted in NaCRRI and NgeZARDI, respectively. The total HCA esters were in the range of 16.9, 18.9, and 56.4 ng/g) at NgeZARDI, NaCRRI, and NaSARRI, respectively.

## Discussion

Recent studies have attempted to understand how insect resistance in crops segregates and whether phenotypic traits are associated with resistance (O’Reilly-Wapstra et al., 2005; Mohammed et al., 2016). Here for the first time we report that HCA esters confer resistance to SPW in sweetpotato, these constituents segregate in the F1, and they are correlated with resistance in both the field and laboratory suggesting that HCA’s might be useful as markers for resistance in sweetpotato breeding programs. Data for some F_1_ genotypes in this study indicated that resistance could be enhanced. There were significant differences in the SPW infestations among the genotypes of the segregating population (**Figure 1**). Twenty five genotypes recorded low storage root damage. Mean SPW storage root infestation was significantly higher in the experiment planted in November than that planted in June. This might be explained by the fact that the second season experiment planted toward the beginning of the dry season encountered drought stress earlier in the crop’s growth cycle, which resulted in poor crop establishment but also reduced plant resilience to SPW attack with similar effects recorded on the storage roots (Stathers et al., 1999).

It is well known that SPW damage escalates in sweetpotato fields during the dry spells (Stathers et al., 2003). Our results agree with this finding as in the three locations where field experiments were conducted, NaSARRI, located in the drier semi-arid zone had the highest mean weevil root infestation (**Figure 2**). Typically, at NaSARRI dry spells begin at the end of the growing season and are characterized by high levels of soil cracks on the sweetpotato mounds or ridges as the storage roots mature. Jackson et al. (2012) reported a similar finding and argued that storage roots exert tremendous pressure in the soil as they expand during growth. The expansion causes soil to crack thereby creating entry avenues for gravid SPW’s to access the storage roots to lay eggs.

This study also found significant genotype × environment (G × E) interaction effects on SPW damage indicating that SPW damage is influenced by environmental effects and that breeding progenies should be tested at multiple sites and seasons while selecting for resistance (Stathers et al., 1999; Grüneberg et al., 2010). There was a significant correlation between field stem infestation and field storage root infestation among the genotypes corresponding with earlier suggestions that stem damage can be an indicator for screening sweetpotato varieties for resistance to SPW (Muyinza et al., 2007) and (**Table 2**) which otherwise requires uprooting plants.

**Table 2:** Correlations between sweetpotato weevil damage in the field, in laboratory studies and concentration of hydroxycinnamic acid esters for a segregating population of New Kawogo and Beauregard.

Sweetpotato damage parameters	Total hydroxycinnamic acid esters concentration	Field root infestation	Field stem infestation
Field root infestation (*n* = 852)	0.603 (*P* ≤ 0.0105)		
Field stem infestation (*n* = 1704)	-0.618 (*P* ≤ 0.05)	0.497 (*P* ≤ 0.001)	
Mean number of feeding holes in lab study (*n* = 1734)	-0.64 (*P* ≤ 0.05)	0.501 (*P* ≤ 0.05)	0.405 (*P* ≤ 0.05)

During drought, soil water is low so less water will be absorbed and transported throughout the plant leading to wilting (Mao et al., 2004). These conditions exacerbate SPW infestation and the transportation of plant defense phytochemicals throughout the plant may be hindered due to the reduced transport of metabolites flowing through the vascular tissues (Ni et al., 2009).

New Kawogo was reported to have high levels of field resistance (Muyinza et al., 2012), whereas Beauregard was highly susceptible to a host of insect pests including SPW (Rolston et al., 1987) and (**Figure 1**). The high level of genetic diversity observed in the New Kawogo × Beauregard population reported here supports the observation of some progeny exhibiting transgressive segregation for resistance to SPW. Variation in SPW infestation observed in the F_1_ population of these two parents was significant and transgressive segregation for SPW resistance was observed in the F_1_ sibs (Yada et al., in press). The progeny that was more resistant than New Kawogo and others with high HCA esters are new candidates for selection and can be used as sources of genes for future crop improvement (**Figure 4**). Grüneberg et al. (2009) suggested that variation in progeny from a bi-parental cross is attributed to the diverse nature of the parents selected from the heterotic gene pool suggesting occurrence of transgressive segregation.

The significant negative correlation between the number of feeding holes in laboratory studies and total HCA ester concentrations on the storage root surface indicated that varieties with higher concentrations of these compounds deter feeding by SPW thereby conferring resistance in sweetpotato (**Table 2**). The significant differences in the total HCA esters among the genotypes of the segregating population could be attributed to genetic effects at multiple loci in the sweetpotato genome for this trait in the cross. Previous studies have shown that New Kawogo (female) has a high concentration of HCA esters on the root surface and high field resistance to SPW in earlier studies (Muyinza et al., 2012; Anyanga et al., 2013). HCA esters were also reported to reduce feeding and oviposition in *C. puncticollis* and *C. brunneus* in feeding and oviposition bioassay by Stevenson et al. (2009). Furthermore, HCA esters were reported to have an effect on the mortality of SPW on artificial diets, which is the reason they were hypothesized to be the chemical basis of resistance in New Kawogo to SPW (Stevenson et al., 2009; Anyanga et al., 2013).

Hydroxycinnamic acid esters have been shown in other studies by Boerjan et al. (2003) to play key roles in the biosynthesis pathway of lignin, a key mode of plant defense against pathogenic attack and herbivory. The pathway of formation of HCA is reported to be catalyzed by the phenylalanine ammonia lyase (PAL), a widely distributed enzyme present in the plant kingdom (Hyun et al., 2011). Vogt (2010) indicated that this enzyme deaminates L-phenylalanine to yield (*E*)-cinnamic acid, which undergoes other enzymatic transformations, yielding a diversity of related products. The significant negative correlation between SPW storage root infestation and total HCA ester concentration (**Table 2**) relates to this finding. Five genotypes (NKB152, NKB257, NKB108, NKB59, and NKB60) showed high and consistent performance on the basis of sweetpotato weevil root infestation and total HCA ester concentrations. These clones could be further evaluated for selection and use as breeding lines for sweetpotato weevil resistance.

Significant positive correlation between the mean numbers of sweetpotato weevil feeding holes, field root infestation and field stem infestation (**Table 2**) indicates that the occurrence of HCAs in higher concentrations on the root surface contributed to the resistance observed in New Kawogo. The low mean number of *C. puncticollis* feeding holes on clones NKB152, NKB257, NKB72, NKB59, NKB260, NKB225, NKB108, NKB52, NKB158, and NKB279 was consistent with low sweetpotato weevil damage in the field indicating that there was transgressive segregation for SPW resistance. Field resistance to SPW was significantly influenced by environment; therefore further evaluation of this population should be undertaken in multi-location sites with high populations for SPW resistance. The transgressive segregating clones need to be selected and screened further to confirm the level of SPW resistance for use in population improvement. Even when a resistant variety is developed it will be important to support this by integrating alongside additional crop management practices to provide a full package for development of sweetpotato weevil resistance for sustainable management of the pest.

## Author Contributions

MA, BY, GY, and PS designed the study. RM started the research which led to this and other studies. MA, BY, and AA carried out the field trials. DF and MA carried out the chemical analysis with guidance from PS and GY. MA ran the bioassay while BY, GY, GS, and RM interpreted the segregation pattern of the progenies. MA, PS, and DF interpreted the chemistry data. MA, BY, GY, PS, and AA wrote the manuscript with significant editorial contributions from GS and RM.

## Conflict of Interest Statement

The authors declare that the research was conducted in the absence of any commercial or financial relationships that could be construed as a potential conflict of interest.
